# Increased IL-6 expression precedes reliable viral detection in the rhesus macaque brain during acute SIV infection

**DOI:** 10.1172/jci.insight.152013

**Published:** 2021-10-22

**Authors:** Raja Mohan Gopalakrishnan, Malika Aid, Noe B. Mercado, Caitlin Davis, Shaily Malik, Emma Geiger, Valerie Varner, Rhianna Jones, Steven E. Bosinger, Cesar Piedra-Mora, Amanda J. Martinot, Dan H. Barouch, R. Keith Reeves, C. Sabrina Tan

**Affiliations:** 1 Center for Virology and Vaccine Research, Department of Medicine, Beth Israel Deaconess Medical Center, Harvard Medical School, Boston, Massachusetts, USA.; 2 Emory Vaccine Center and Yerkes National Primate Research Center, Emory University, Atlanta, Georgia, USA.; 3 Department of Pathology and Laboratory Medicine, Emory University School of Medicine, Atlanta, Georgia, USA.; 4 Department of Comparative Pathobiology, Section of Pathology, and Departments of Infectious Diseases and Global Health and Biomedical Sciences, Tufts University Cummings School of Veterinary Medicine, North Grafton, Massachusetts, USA.; 5 Ragon Institute of Massachusetts General Hospital, MIT, and Harvard, Cambridge, Massachusetts, USA.; 6 Division of Infectious Diseases, Department of Medicine, Beth Israel Deaconess Medical Center, Harvard Medical School, Boston, Massachusetts, USA.

**Keywords:** AIDS/HIV, Neuroscience, Adaptive immunity, Cytokines, Macrophages

## Abstract

Knowledge of immune activation in the brain during acute HIV infection is crucial for the prevention and treatment of HIV-associated neurological disorders. We determined regional brain (basal ganglia, thalamus, and frontal cortex) immune and virological profiles at 7 and 14 days post infection (dpi) with SIV_mac239_ in rhesus macaques. The basal ganglia and thalamus had detectable viruses earlier (7 dpi) than the frontal cortex (14 dpi) and contained higher quantities of viruses than the latter. Increased immune activation of astrocytes and significant infiltration of macrophages in the thalamus at 14 dpi coincided with elevated plasma viral load, and SIV colocalized only within macrophages. RNA signatures of proinflammatory responses, including IL-6, were detected at 7 dpi in microglia and interestingly, preceded reliable detection of virus in tissues and were maintained in the chronically infected macaques. Countering the proinflammatory response, the antiinflammatory response was not detected until increased TGF-**β** expression was found in perivascular macrophages at 14 dpi. But this response was not detected in chronic infection. Our data provide evidence that the interplay of acute proinflammatory and antiinflammatory responses in the brain likely contributed to the overt neuroinflammation, where the immune activation preceded reliable viral detection.

## Graphical abstract

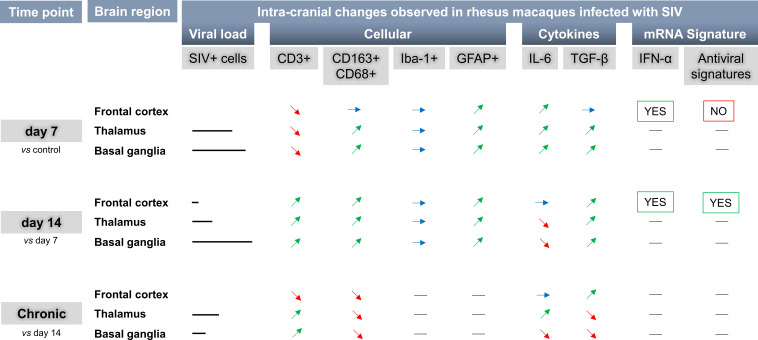

## Introduction

Despite the success of antiretroviral therapy, people with HIV still experience some form of neurocognitive impairment (1). HIV infiltrates the CNS within the first 2 weeks of peripheral infection, even at times without clinically apparent acute neurological manifestations (2–4), and does not necessarily correlate with plasma viral loads. Peripherally, CD8^+^ T cells exert some control of viral infection by cytotoxicity-mediated killing (5). In one study, these cytotoxic CD8^+^ and helper CD4^+^ T lymphocytes independently correlated directly with HIV RNA levels in cerebrospinal fluid (CSF) and in plasma during acute infection, suggesting that peripheral immune response may modulate CNS viral infection (6). However, the severity of CNS lesions did not correlate with the corresponding plasma viral load, despite the correlation between CSF and plasma viral loads (6–8). These data suggest that distinct viral immune responses may be compartmentalized in the CNS. Thus, better understanding of HIV pathogenesis and the associated myeloid or lymphoid cell dynamics in the CNS during acute infection will elucidate factors leading to viral persistence in the brain (9).

Proinflammatory responses in the brain may prevent uncontrolled viral replication in acute lentiviral infection in the brain, but antiinflammatory responses are also crucially needed to preserve brain functions. The timing of these opposing responses in acute infection could influence virus survival of initial immune responses. Prior studies of immune and inflammatory responses in the brain have largely been carried out in nonhuman primates (NHPs) rather than humans owing to relative ease of access to NHP brain tissue, especially for consistent and more reliable longitudinal analysis. SIV-infected rhesus macaque models largely recapitulate HIV viral kinetics, persistence, reservoir formation, and progression to terminal disease (10, 11). Unfortunately, very few studies have characterized the dynamics of immune cells and proinflammatory and antiinflammatory factors in early acute SIV infection in the CNS. Prior studies on acute inflammatory responses in the CNS examined peripheral viral load in conjunction with either histopathological consequences or expression profiles of different inflammatory/antiinflammatory cytokine and chemokine markers in the CNS using CD8^+^ T cell–depleted macaque (13) or simian-human immunodeficiency virus–infected (SHIV-infected) macaques (14), or the accelerated CNS encephalitis in a pigtail macaque model infected with the swarm of SIV/Deltab670 and SIV/17EFr clones (15). NHP models with immune modulations cannot completely decipher the role of cytotoxic CD8^+^ T cells and the usage of SHIVs, and the accelerated infection models do not fully represent acute events of HIV pathogenesis in humans.

Thus, we wanted to thoroughly characterize brain factors associated with acute SIV infection using a non–immune-modulated host infected with standard SIV to model acute CNS infection in HIV (16–19). SIV_mac239_ mimics the pathogenicity of HIV and is highly neutralization resistant, enabling it to cause infection and pathogenesis uniformly, making it a favorable choice for studying early SIV infection and viral reservoir formation (16). In this study, we analyzed the brains of SIV-infected rhesus macaques at 2 time points (7 and 14 days post infection [dpi]) to better understand the immune activation, immune cell dynamics, and inflammatory responses in the brain at these very early time points.

## Results

### Early acute SIV_mac239_ infection in the plasma and 3 anatomical regions of the brain of the rhesus macaque.

SIV plasma viral load was detectable in all 3 macaques euthanized at 7 dpi (*n* = 3) with a mean of 4.99 ± 0.98 log_10_ SIV Gag RNA copies/mL (range = 4.31–6.12), and in all 5 macaques euthanized at 14 dpi with a mean of 6.82 ± 1.484 log_10_ SIV Gag RNA copies/mL (range = 4.38–8.11; Table 1). However, the viral loads in the frontal cortex of the brain were detected at a much lower level (Table 1). At 7 dpi, the frontal cortex did not have detectable levels of SIV Gag DNA or RNA, with the exception of 1 animal (Rh4), which had 228 SIV Gag DNA copies per 1 million cells. In contrast, all the animals at 14 dpi had detectable viral DNA and RNA in the frontal cortex, though at varying levels (Table 1).

**Figure 1:**
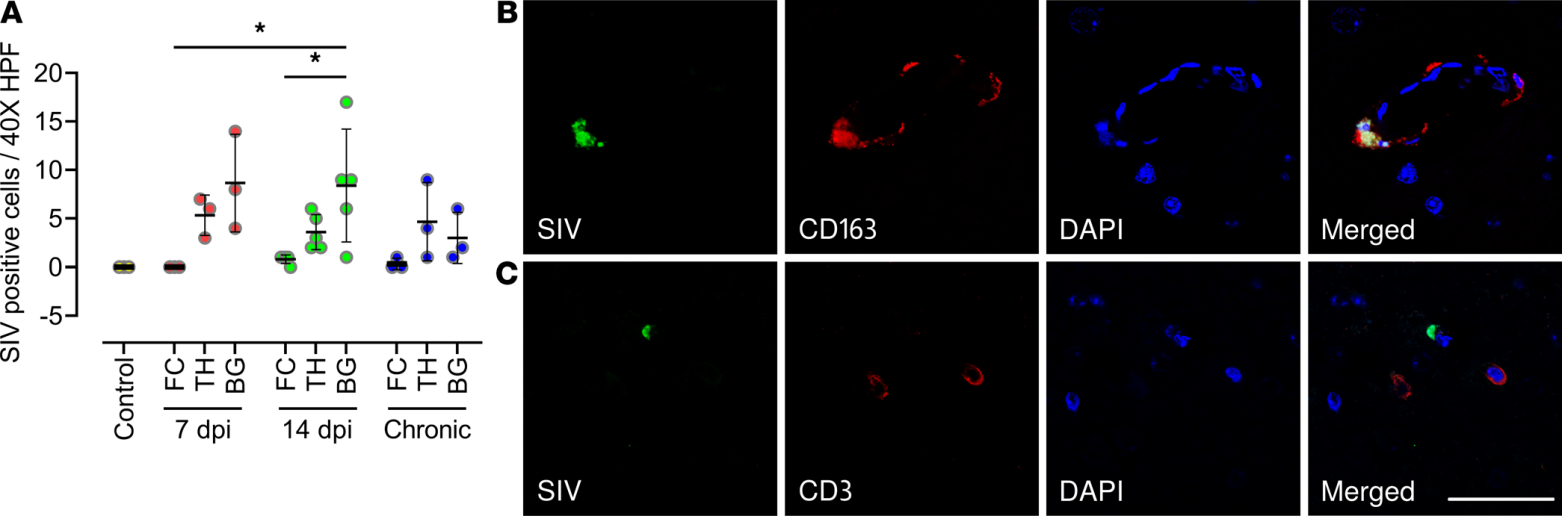
Quantitation of SIV viral RNA^+^ cells in the macaque brain and colocalization of SIV with perivascular macrophages. (**A**) SIV viral RNA^+^ cells in frontal cortex, basal ganglia, and thalamus of uninfected control, acutely infected (7 and 14 dpi), and chronically infected macaque brains were detected by combining RNAscope with immunofluorescence and counted manually by visualizing slides under a light microscope. Each data point indicates 1 animal, and the associated numerical value is the average number of SIV^+^ cells counted in 15 high-powered fields (HPFs) at 40× under light microscope. (**B** and **C**) SIV in frontal cortex of macaque brain colocalized with perivascular CD163^+^ macrophages but not with CD3^+^ cells. RNAscope SIV-probe conjugated with FITC (green) colocalized with perivascular macrophages (red) stained with Alexa Flour 647–conjugated CD163 antibodies (**B**) and did not colocalize with CD3^+^ cells (red) stained with Alexa Fluor 647–conjugated CD3 antibodies (**C**). Data are presented as mean ± SD. Statistical significance was calculated using a nonparametric Kruskal-Wallis test and multiple comparisons were assessed using Dunn’s post hoc analysis. *P ≤ 0.05. *n* = 3 (control, 7 dpi, chronic), *n* = 5 (14 dpi) (**A**). Scale bar: 50 μm (**B** and** C**).

To evaluate potential regional differences in virus replication in the brain, we also quantified SIV in the basal ganglia and thalamus in addition to the frontal cortex. Viral DNA and RNA in Rh11 (14 dpi) were of comparable quantities in all 3 brain regions by RT-qPCR (Table 1). Viral RNA, in all 3 brain regions, was further localized and quantified by in situ hybridization (ISH) using RNAscope (Figure 1A). Among the 3 animals at 7 dpi, there was no detectable SIV RNA in the frontal cortex sections examined, similar to our findings using RT-qPCR above, while the basal ganglia and thalamus regions had few positively stained cells for SIV RNA. In contrast, frontal cortex sections from all animals at 14 dpi, except one, had cells containing detectable SIV RNA. Both at 7 (*P* = 0.045) and 14 dpi (*P* = 0.010), the number of SIV^+^ cells, was significantly higher in the basal ganglia when compared with the frontal cortex (Figure 1A). In addition, we also quantified SIV RNA by ISH in 3 rhesus macaques infected with SIV for more than 180 days (chronic). The SIV-positive cell counts were not significantly different among the 3 regions of the chronically infected animals or between the chronically infected macaques and macaques at 7 or 14 dpi in any of the given brain regions analyzed. Further characterization of brain cells showed that SIV-positive cells colocalized with CD163^+^ cells, both in the perivascular and parenchymal regions (Figure 1B), and the SIV RNA did not colocalize with CD3^+^ cells in our experiments (Figure 1C).

### Acute SIV_mac239_ infection elicited brain-intrinsic immune activation and infiltration of peripheral immune cells.

**Figure 2:**
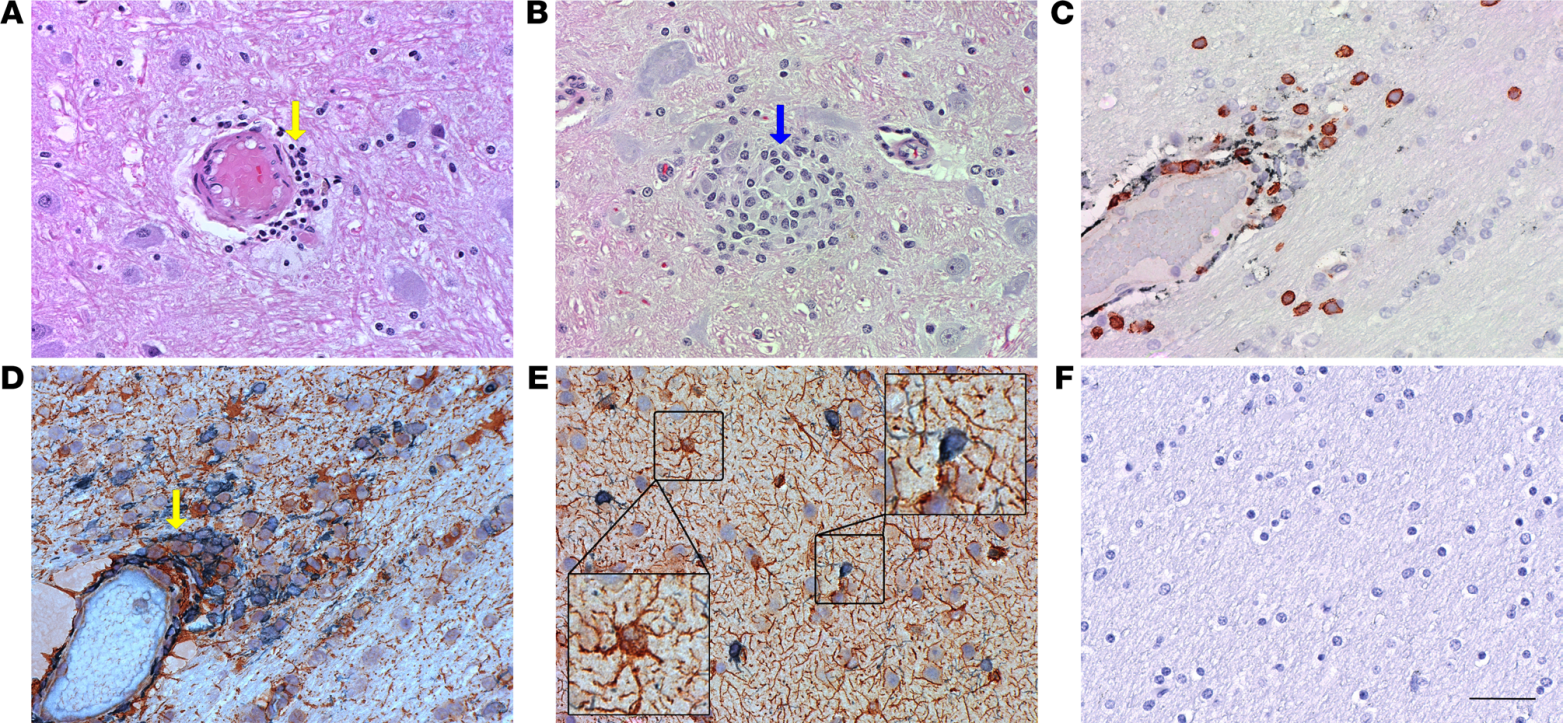
H&E and IHC staining of SIV-infected macaque brain. (**A** and** B**) H&E staining of brain section showed immune cell infiltration (yellow arrow in **A**) and aggregation of macrophages (blue arrow in **B**). (**C**–**E**) IHC staining with lineage markers for T cells (CD3) and macrophages (CD163/CD68) showed most of the infiltrating cells were CD3^+^ cells (brown staining in **C**); the other few were macrophages (gray-black staining in **C**). Further IHC staining for brain-resident immune cells revealed astrocyte-macrophage aggregation (yellow arrow in **D**). Astrocyte activation (right inset in **E**) and rare instances of microglial (Iba-1) activation (left inset in **E**) were also seen. (**F**) Isotype control used for IHC staining in **C**–**E**. Slides were counterstained with hematoxylin after IHC staining. Scale bar: 50 μm.

To understand the pathogenesis associated with acute SIV infection in the macaque brains, we examined H&E-stained sections of the basal ganglia, thalamus, and frontal cortex from animals euthanized at 7 and 14 dpi. While we did not find any large areas of neurological or neuropathological abnormalities in the brain parenchyma during acute infection (7 dpi and 14 dpi; Figure 2), we did observe a few areas of increased perivascular localization of immune cells (Figure 2A) and macrophage aggregation (Figure 2B), especially at 14 dpi. Further characterization of infiltrating immune cells using IHC revealed that most of the infiltrating immune cells on 14 dpi were CD3^+^ lymphocytes and few of them were CD163^+^/CD68^+^ macrophages (Figure 2C). IHC staining for brain-resident cells revealed astrocyte-macrophage aggregation around the blood vessels (Figure 2D). In addition to astrocyte activation (extensive branching) in the parenchymal regions, we also observed microglial activation (shortened processes), which was not seen in the IgG negative control (Figure 2, E and F).

**Figure 3:**
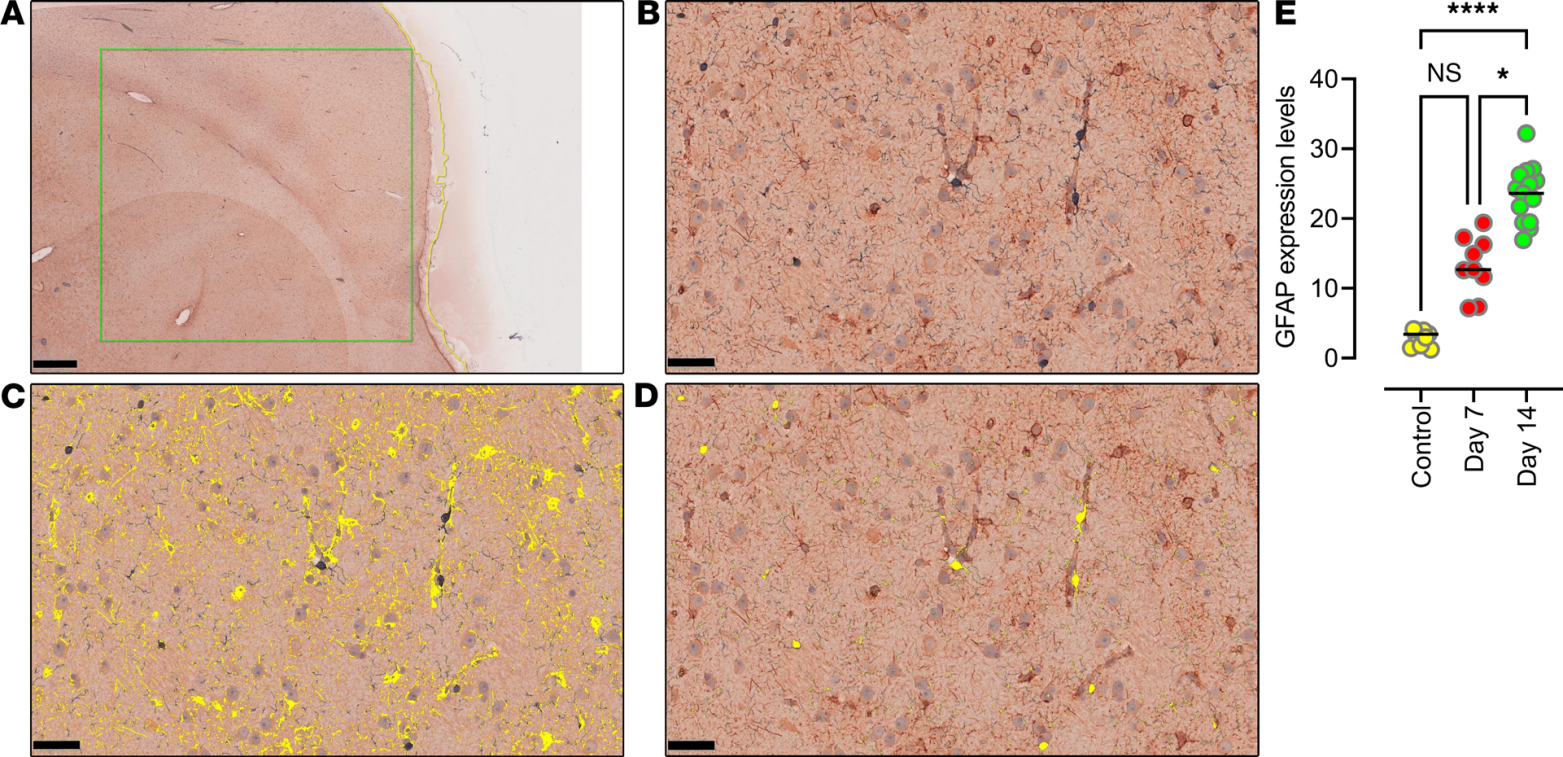
Quantitative determination of GFAP and Iba-1 expression using tissue image analysis. (**A**–**E**) Quantitative expression analysis of GFAP and Iba-1 was performed by scanning a representative 50 mm^2^ area in the frontal cortex histological section immunostained for GFAP and Iba-1 markers. Microphotograph of a histological section of the frontal cortex (**A**) chosen for multiplex IHC showing positive immunostaining represented by DAB staining for GFAP and blue chromogen staining for Iba-1 (**B**). By markup highlighting the positively stained areas for GFAP (**C**) and Iba-1 (**D**), quantitative analysis was carried out using HALO software (v2.3.2089.27 or v3.0.311.405; Indica Labs). The graphical representation of significantly increased GFAP expression levels at 14 dpi when compared with control and 7 dpi (**E**). Scale bars: 1 mm (**A**) and 50 μm (**B**–**D**). Data are presented as mean ± SD. Statistical significance was calculated using a nonparametric Kruskal-Wallis test and multiple comparisons were assessed using Dunn’s post hoc analysis. *P ≤ 0.05, ****P ≤ 0.0001. *n* = 3 (control, 7 dpi), *n* = 5 (14 dpi) (**E**).

To quantify changes in the astrocyte population, we used HALO image analysis software and determined that the glial fibrillary acidic protein (GFAP) expression levels in the 3 brain regions combined were significantly higher at 14 dpi than the comparable brain regions of uninfected control animals (*n* = 3; *P* < 0.0001) and 7 dpi (*P* = 0.014), indicating astrocyte activation with infection (Figure 3, A–C, and E). Among the 3 brain regions analyzed, the thalamus region had significantly higher expression of GFAP at both 7 dpi and 14 dpi, while the frontal cortex had the lowest, indicating that increased immune activation may correlate to quantities of virus detected in the tissues. However, tissue image analysis of microglia expression levels of ionized calcium-binding adaptor molecule-1 (Iba-1) did not show significant differences in either of the infected groups as compared with the uninfected control animals (Figure 3D).

### Upregulation of proinflammatory IL-6 during early acute infection was expressed by microglia in the brain.

**Figure 4:**
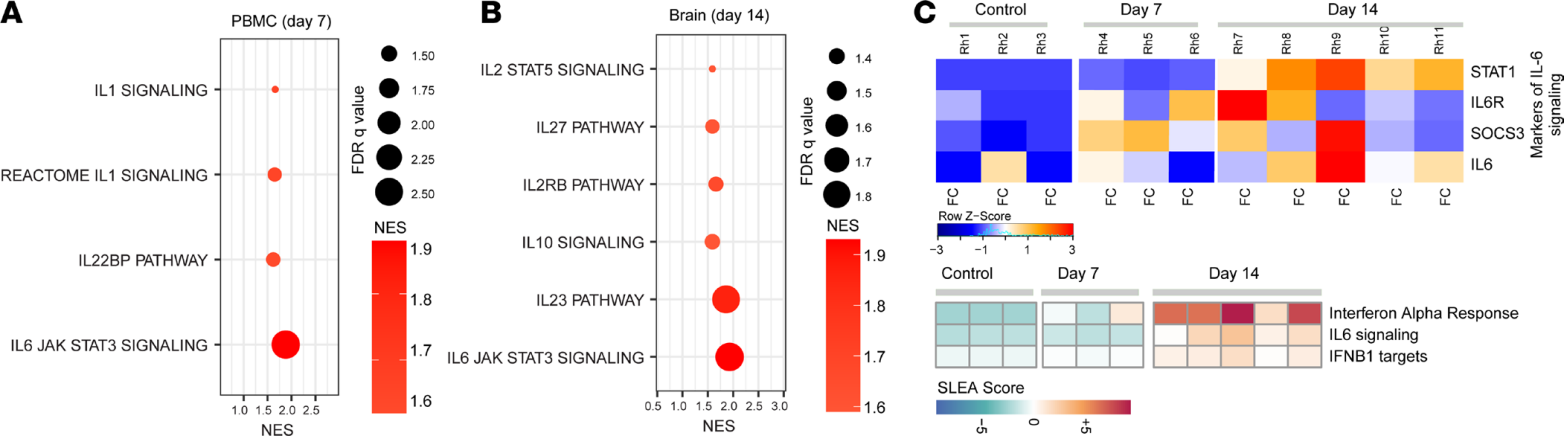
Expression of proinflammatory cytokines in the brain of SIV-infected macaques. (**A** and** B**) Pathway enrichment analysis of signatures of cytokine signaling in PBMCs (**A**) and in the frontal cortex (FC) (**B**) of SIV-infected macaques at 7 and 14 dpi. Cytokine signaling signatures at 14 dpi (changes not significant compared with 7 dpi) in PBMCs and 7 dpi (changes not significant compared with control) in FC were not included. Circle size is proportional to the FDR *q* value of each pathway as measured by GSEA. Circle color corresponds to pathway normalized enrichment score (NES) at 7 and 14 dpi compared with uninfected control macaques. (**C**) Top panel: heatmap of the leading-edge genes that contribute to the enrichment of IL-6 signaling pathways in the FC of macaque brain at 7 and 14 dpi compared with the uninfected control. Each row represents normalized mRNA expression of a gene across all 7 and 14 dpi and chronic animals. Each column represents an animal belonging to control group (first bloc) and at day 7 (second bloc) and day 14 (third bloc) after SIV infection. Changes in the row *z* score are represented as unique color from lowest (blue) to highest (red) color gradient; lower panel: SLEA score of pathways of IFN-α response, IL-6 signaling, and IFNβ1 targets at 7 and 14 dpi in the brain compared with control macaques. *n* = 3 (control, 7 dpi), *n* = 5 (14 dpi) (**C**).

At 7 dpi and 14 dpi, we analyzed the inflammatory and antiinflammatory cytokine response of immune cells during acute SIV infection by RNA-Seq of PBMCs and the frontal cortex of all infected animals, as well as the thalamus and basal ganglia of Rh11. A significant upregulation of proinflammatory pathways, such as IL-6/JAK/STAT3 and IL-1 signaling (Figure 4A), was detected as early as 7 dpi in the PBMCs. In the frontal cortex of macaques, we observed upregulation of cytokine signaling pathways, including IL-6, IL-10, and IL-27, at 14 dpi (Figure 4B). The heatmap of the leading-edge genes that contributed to the enrichment of the IL-6 signaling pathways was found to be enriched at 7 dpi with further enhancement at 14 dpi in the macaque brain (Figure 4C).

**Figure 5:**
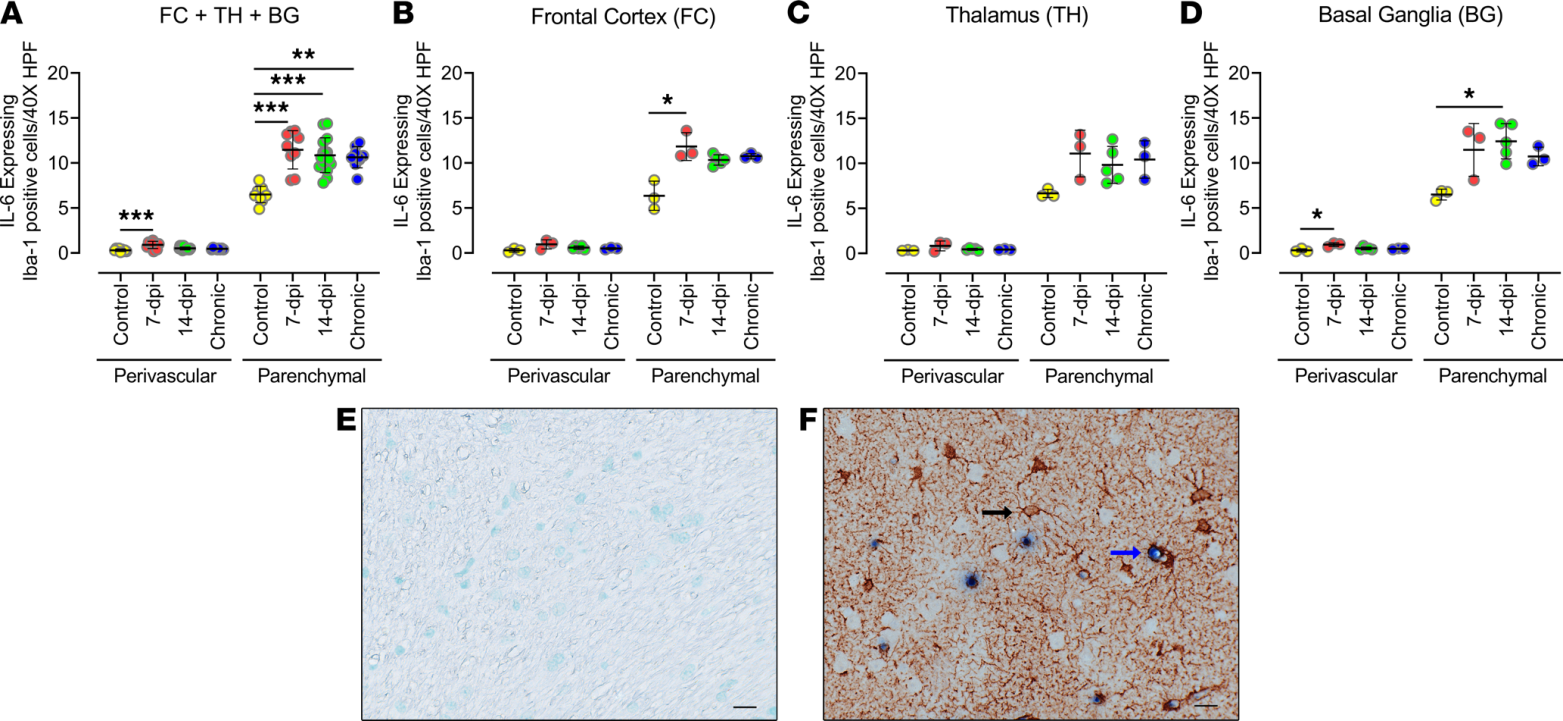
Quantities of IL-6–expressing microglia cells increased with acute infection and remained elevated in chronically infected animals. (**A**–**D**) IL-6–producing cells in frontal cortex, thalamus, and basal ganglia of macaque brain after infection with SIV were identified using anti–IL-6 antibody costained (double positive) with antibodies against Iba-1. Analysis of double-positive cells in the 3 brain regions combined (**A**) and in individual brain regions: frontal cortex (**B**), thalamus (**C**), and basal ganglia (**D**). Each data point indicates 1 animal, and the associated numerical value is the average number of cells counted in 15 HPFs at 40× under light microscope. (**E** and **F**) IHC images of negative control (**E**) and positive staining (single stained for Iba-1 in brown [black arrow]; double stained [blue arrow] for Iba-1 and IL-6 in brown and blue, respectively) (**F**). Data are presented as mean ± SD. Statistical significance was calculated using a nonparametric Kruskal-Wallis test and multiple comparisons were assessed using Dunn’s post hoc analysis. *P ≤ 0.05, ***P ≤ 0.001. *n* = 3 (control, 7 dpi, chronic), *n* = 5 (14 dpi) (**A**–**D**). Scale bar: 20 μm (**E** and** F**).

To better assess the cells that express IL-6 during acute infection, we examined the brain tissues by IHC and found that IL-6 did not colocalize with CD3^+^, GFAP^+^, or CD163^+^/CD68^+^ cells. Instead, IL-6 expression was detected in a subgroup of Iba-1^+^ cells (Figure 5). Analysis of IL-6–expressing Iba-1^+^ cells in all 3 brain regions combined revealed that the number of Iba-1^+^ cells expressing IL-6 in the parenchyma of the brain tissue had significantly increased at 7 dpi (*P* = 0.0002), 14 dpi (*P* = 0.0007), and in the chronic group (*P* = 0.0063) compared with uninfected controls, and there were no significant differences between these 3 infected groups (Figure 5A). A similarly increased colocalization was observed in the perivascular location, but the increase was significant only at 7 dpi. Individually, all 3 brain regions exhibited a similar response with a significant increase in IL-6–expressing Iba-1^+^ cells in the parenchyma of the frontal cortex at 7 dpi (*P* = 0.0188) and in the parenchyma of the basal ganglia at 14 dpi (*P* = 0.0477; Figure 5, B–D). Of note, the parenchymal location also contained a higher number and percentage of IL-6–expressing Iba-1^+^ cells (Figure 5, E and F). Although our tissue image analysis above did not detect a significant increase in Iba-1 expression in the infected groups as compared with the uninfected control group, RNA-Seq analysis did detect upregulation of microglial activation gene signatures as early as 7 dpi in the frontal cortex (Supplemental Figure 1). Furthermore, the microglial gene signatures were more upregulated in the thalamus and basal ganglia of macaque Rh11 than in its frontal cortex. These data indicate that microglia produced IL-6 in early acute SIV infection, even in tissues without detectable virus, and this expression level was maintained in our macaques infected with SIV for more than 180 days.

### Upregulation of the antiinflammatory response includes expression of TGF-β expressed by macrophages.

**Figure 6:**
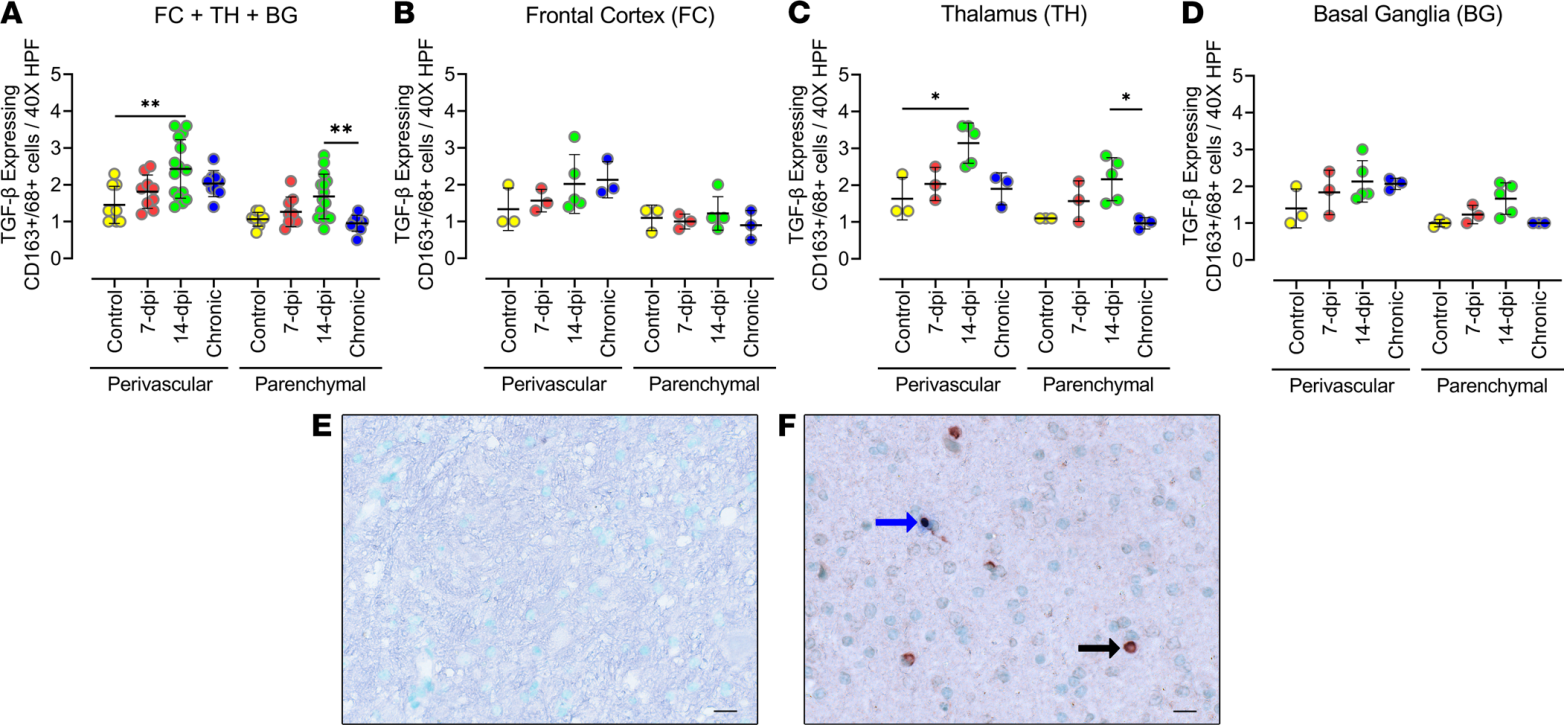
Quantities of TGF-β–expressing CD163^+^/CD68^+^ cells increased with acute infection but not sustained in the chronically infected animals. (**A**–**D**) Cells producing TGF-β in the frontal cortex, thalamus, and basal ganglia of macaque brain, after infection with SIV, were identified using anti–TGF-β antibodies costained with antibodies against macrophage lineage cells (CD163^+^/CD68^+^). Analysis of double-positive cells in the 3 brain regions combined (**A**) and in individual brain regions: frontal cortex (**B**), thalamus (**C**), and basal ganglia (**D**). Each data point indicates 1 animal, and the associated numerical value is the average number of cells counted in 15 HPFs at 40× under light microscope. (**E** and **F**) IHC images of negative control (**E**) and positive staining (single stained for CD163^+^/CD68^+^ cells in brown [black arrow]; double stained [blue arrow] for CD163^+^/CD68^+^ cells and TGF-β in brown and blue, respectively (**F**). Data are presented as mean ± SD. Statistical significance was calculated using a nonparametric Kruskal-Wallis test and the multiple comparisons were assessed using Dunn’s post hoc analysis. *P ≤ 0.05, **P ≤ 0.01. *n* = 3 (control, 7 dpi, chronic), *n* = 5 (14 dpi) (**A**–**D**). Scale bar: 20 μm (**E** and** F**).

To characterize antiinflammatory responses in the brain, we examined the enrichment of several TGF-β signaling pathways at 7 and 14 dpi in the frontal cortex of the macaque brain. Although gene set enrichment analysis (GSEA) did not reveal a significant (nominal *P* < 0.05) enrichment of these pathways, we detected a subset of TGF-β signaling genes that were increased in the brain of infected macaques compared with control animals that included SMAD7, SMAD6, and TGFB1 on 7 and 14 dpi (Supplemental Figure 2). We examined different cells for TGF-β expression in the brain tissue and determined that the expression did not colocalize with cells positive for CD3^+^, Iba-1, or GFAP. TGF-β expression was observed in a subgroup of CD163^+^/CD68^+^ cells (Figure 6). Unlike IL-6–expressing cells, there was no notable difference in the number of TGF-β–expressing cells between the perivascular and parenchymal locations (Figure 6, A–D). But there was a stark difference in the percentage of TGF-β–expressing CD163^+^/CD68^+^ cells between the perivascular (50%–90%) and parenchymal locations (6%–12%). Combining all 3 brain regions, there was a significant increase in TGF-β–expressing cells at 14 dpi compared with uninfected controls in the perivascular location (Figure 6A). In the individual brain regions, the thalamus showed a significantly increased number of TGF-β–expressing cells in the infected animals when compared with the uninfected control; the basal ganglia exhibited a trend of increasing TGF-β–expressing cells, whereas the frontal cortex did not show any remarkable change in TGF-β–expressing cells (Figure 6, B–D). We observed that only the perivascular location of the thalamus (*P* = 0.0411) had a significant increase in macrophages expressing TGF-β at 14 dpi compared with uninfected controls (Figure 6, C–F). Chronically infected animals showed significantly reduced expression of TGF-β in macrophages when compared with animals at 14 dpi (*P* = 0.035) in the parenchyma of the thalamus. These data showed a slight delay in the increase of TGF-β expression in macrophage cells migrating into the brain, and the increased expression was not maintained in chronic infection.

### Distinct immune cell activation profiles in the macaque brain as compared with PBMCs characterized by differing macrophage polarization states.

**Figure 7:**
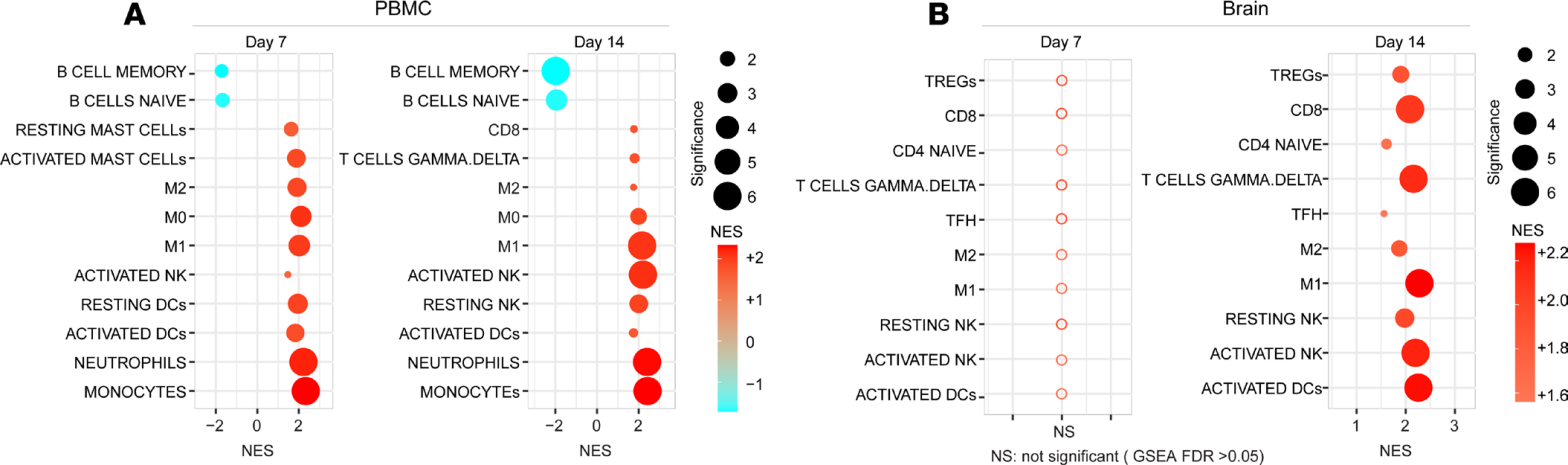
Delayed expression of immune cells activation markers in SIV infected macaque brain. Pathway enrichment analysis of signatures of innate and adaptive immune cells in PBMCs (**A**) and in the brain (**B**) of SIV infected macaque at 7 and 14 dpi. Circle size is proportional to the GSEA: FDR *q* value of each pathway as measured by GSEA. Circles color corresponds to NES at 7 and 14 dpi compared with uninfected control macaques. Low to high NES score denoted by cyan to red color transition. Empty circles represent nonsignificance (**B**).

In order to better understand the impact of acute SIV_mac239_ infection on the overall immune milieu in the brain, we analyzed and compared RNA-Seq data of immune cell activations in PBMCs and the frontal cortex, using the uninfected control animals as baseline. Additional RNA-Seq analysis on the thalamus and basal ganglia was also performed in Rh11. First, we identified differentially expressed genes (DEGs) in the brain at 7 and 14 dpi compared with the uninfected macaques followed by pathway enrichment analysis using GSEA (20, 21). In the PBMCs of infected macaques, we observed upregulation of signatures of immune populations, including monocytes, T cells, DCs, NK cells, and macrophages (M0, M1, and M2), at 7 and 14 dpi (Figure 7A). In the brain, significant increases in T cell (CD4, CD8), macrophage (M1 and M2), and activated NK pathways were seen at 14 dpi (Figure 7B). The distributions of macrophages among the 3 detected polarization states, M0, M1, and M2, differed in PBMCs between 7 and 14 dpi and between PBMCs and the frontal cortex on 14 dpi. In PBMCs, at 7 dpi, all 3 polarization states had similar significance in pathway enhancement; on 14 dpi the proinflammatory M1 state gene pathway was more significantly enhanced than the antiinflammatory M2 state. In the frontal cortex on 14 dpi, there was no M0 gene signature detected, but the M2 pathway was more enhanced than in PBMCs with a comparable M1 significance. These dynamic changes in the macrophage polarization states may be associated with TGF-β expression.

**Figure 8:**
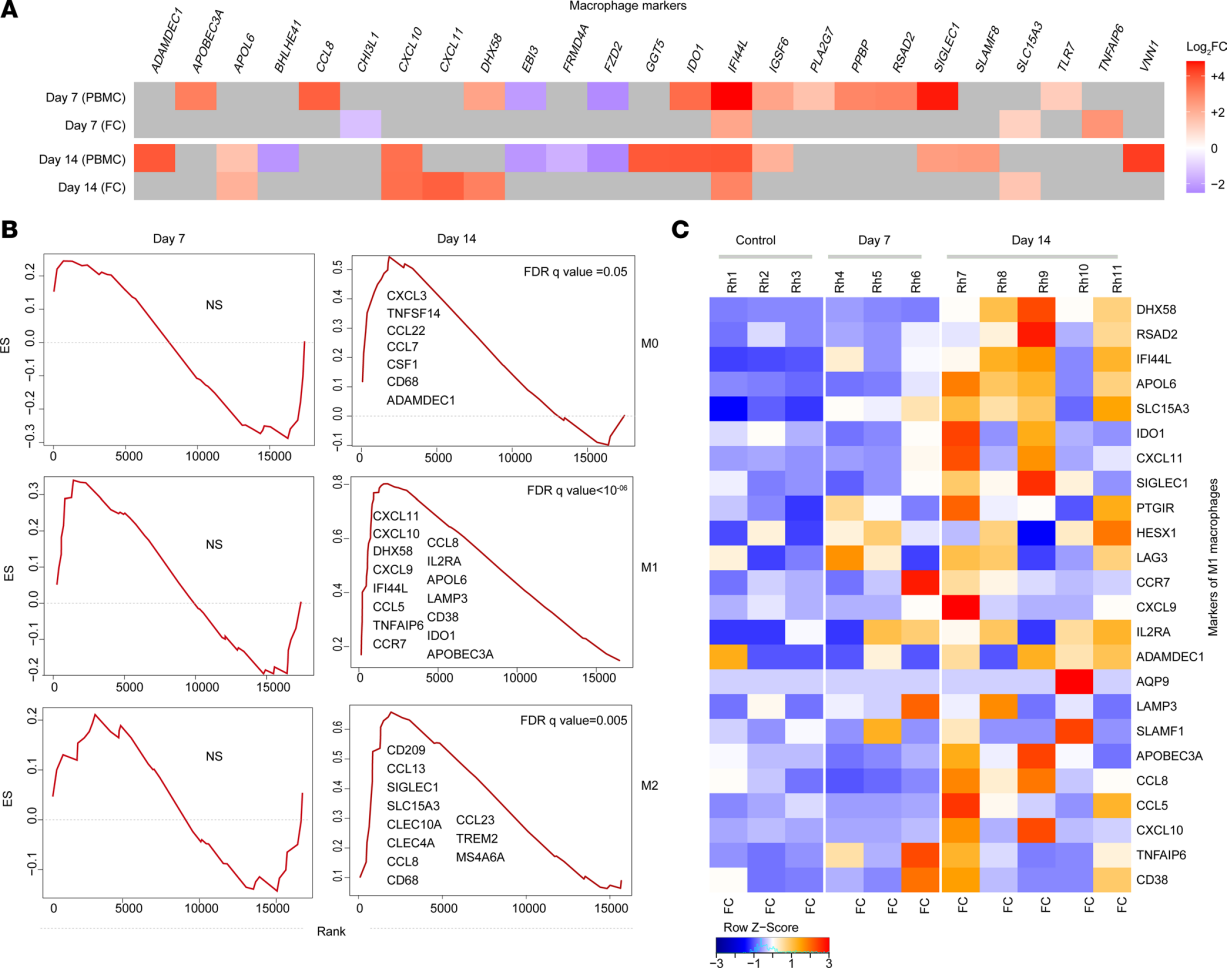
Upregulation of macrophage gene signatures in frontal cortex of SIV-infected macaques. (**A**) Heatmap of the leading-edge genes that contribute to the enrichment of macrophage markers in the PBMCs and frontal cortex (FC) of macaque brains at 7 and 14 days after SIV infection. (**B**) Signatures of macrophages M0, M1, and M2 in the macaque brain at 7 and 14 dpi. (**C**) Heatmap of the leading-edge genes that contribute to the enrichment of M1 macrophage marker in frontal cortex. Each row represents normalized mRNA expression of a gene across all 7 and 14 dpi and chronic animals. Each column represents an animal belonging to control group (first bloc) and at day 7 (second bloc) and day 14 (third bloc) after SIV infection. Log_2_ fold change (FC) gene expression is represented with unique color denoting lowest (blue) to highest (red) color gradient (**A**). NS: signature is not significant (**B**). Changes in the row *z* score are represented as unique color from lowest (blue) to highest (red) color gradient (**C**). *n* = 3 (control, 7 dpi), *n* = 5 (14 dpi) (**C**).

Multiple macrophage activation pathways were detected in the frontal cortex by RNA-Seq (Figure 8A). Among the different macrophage polarization states, gene pathway signatures of the proinflammatory M1 macrophages were found to be more enriched than M2 or M0 macrophages (Figure 8B). The heatmap analysis of the leading-edge genes of proinflammatory M1 macrophages showed the M1 gene signature was upregulated, particularly at 14 dpi (Figure 8C). Finally, we also detected an upregulation of the oxidative phosphorylation gene pathway in the frontal cortex as early as 7 dpi (Supplemental Figure 2), indicating macrophage activation with changing polarization state.

**Figure 9:**
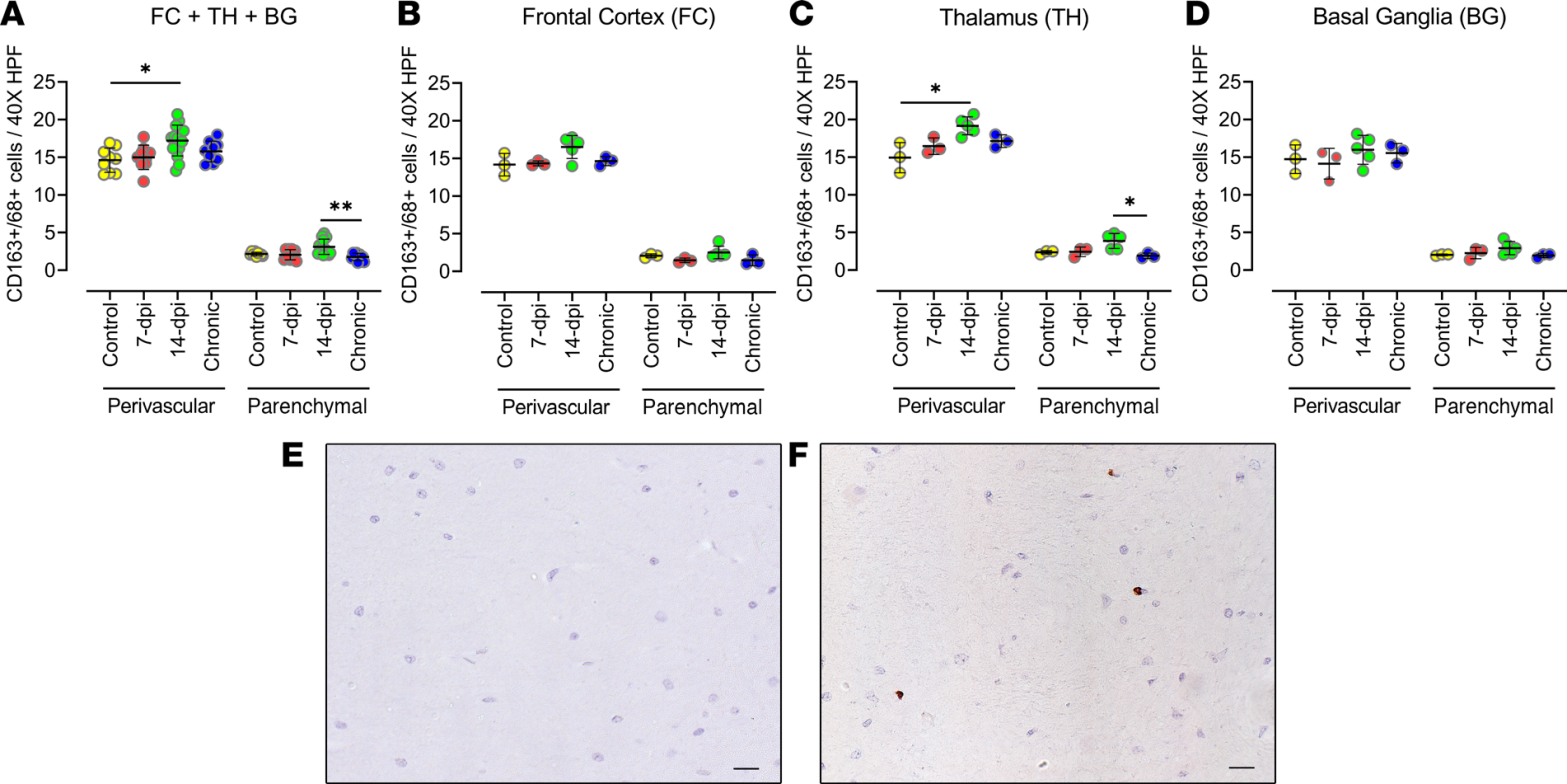
Quantities of macrophage cells increased at 2 weeks after infection in SIV-infected macaque brains. (**A**–**D**) The macrophage lineage (CD163^+^/CD68^+^) cell number change in macaque brains in response to SIV infection (7 and 14 dpi) was studied though IHC staining of the frontal cortex, thalamus, and basal ganglia with anti-CD163/CD68 antibodies and further counting of positive cells manually under light microscope. Analysis of macrophages in the 3 brain regions combined (**A**) and in individual brain regions: frontal cortex (**B**), thalamus (**C**), and basal ganglia (**D**). Each data point indicates 1 animal, and the associated numerical value is the average number of cells counted in 15 HPFs at 40× under light microscope. (**E** and **F**) IHC images of negative control (**E**) and positive staining for macrophages (**F**) (with DAB brown). Data are presented as mean ± SD. Statistical significance was calculated using a nonparametric Kruskal-Wallis test and multiple comparisons were assessed using Dunn’s post hoc analysis. *P ≤ 0.05, **P ≤ 0.01. *n* = 3 (control, 7 dpi, chronic), *n* = 5 (14 dpi) (**A**–**D**). Scale bar: 20 μm (**E** and** F**).

To further determine the presence of macrophages in the brain with SIV infection, we performed IHC analysis of macrophage (CD163^+^/CD68^+^) populations in the 3 brain regions of animals acutely infected (7 or 14 dpi) and chronically infected with SIV (Figure 9). The analysis of the macrophage cell population in the brain, adding the 3 brain regions, showed that in the perivascular location, the quantities of macrophages significantly increased at 14 dpi as compared with the control (*P* = 0.0149), and an increasing trend was observed at 7 dpi (Figure 9A). However, the CD163^+^/CD68^+^ cell population in the chronically infected animals was similar to that of the uninfected control group in all the brain regions individually and in cumulative analysis. In the thalamus, we observed a significant increase in the quantities of CD163^+^/CD68^+^ cells in the perivascular location (*P* = 0.0293) at 14 dpi (Figure 9C). But in the frontal cortex and the basal ganglia, the quantities of the CD163^+^/CD68^+^ cell population at 7 and 14 dpi were not statistically different from uninfected controls (Figure 9, B–F). Thus, the significant increase in macrophage presence in the brain was delayed until 14 dpi, similar to the detection of virus, and the increase was not found in those with chronic infection.

### RNA transcripts of antiviral and IFN signaling pathways detected T lymphocyte activation in the frontal cortex despite rare detection of SIV.

**Figure 10:**
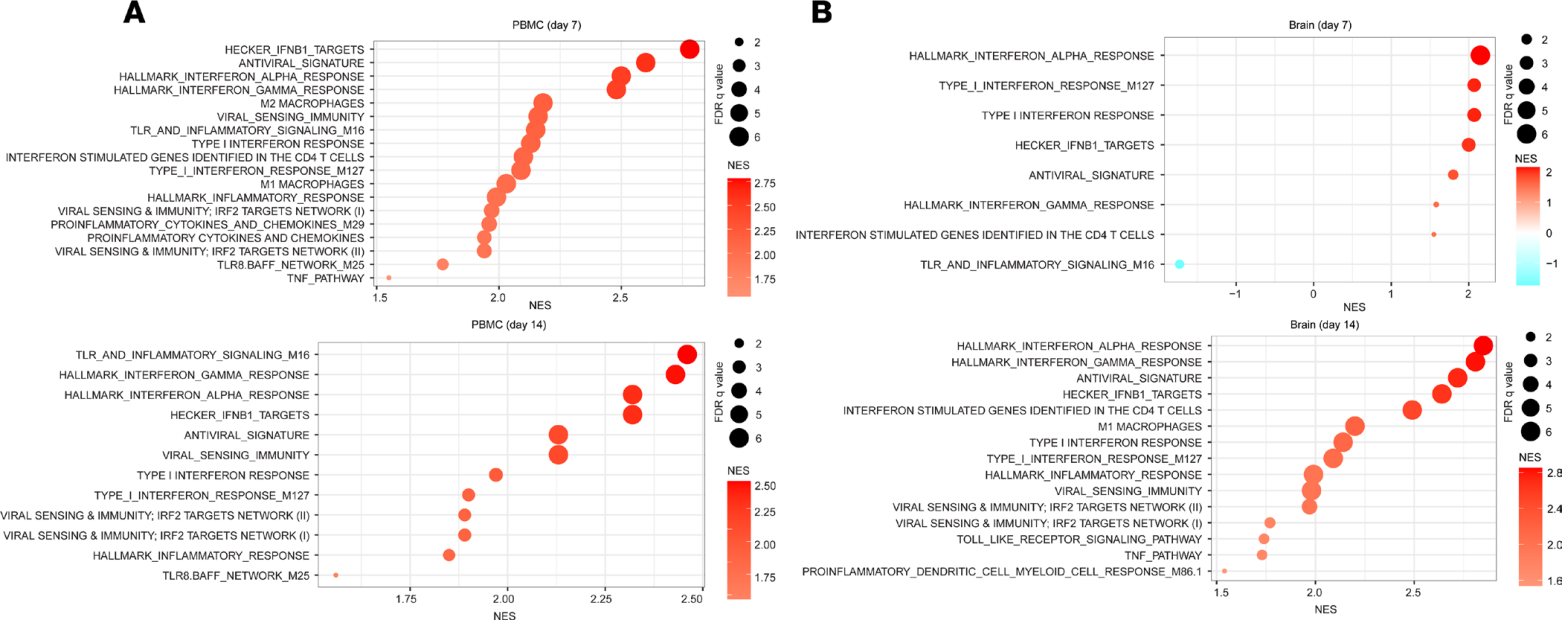
The delayed expression of signatures of antiviral genes and IFN signaling in the brain of infected macaques. Pathway enrichment analysis of signatures of antiviral response and IFN and inflammatory pathways in the PBMCs (**A**) and in the brain (frontal cortex, FC) (**B**) of SIV-infected monkeys at 7 and 14 dpi. Circle size is proportional to the FDR *q* value of each pathway as measured by GSEA. Circle color corresponds to NES at 7 and 14 dpi compared with uninfected control macaques.

In addition to proinflammatory signatures, transcriptomic analyses showed that the antiviral signatures, IFN response, and viral sensing and immunity targets were also significantly upregulated in PBMCs at both 7 and 14 dpi (Figure 10A). In the frontal cortex, only the IFN response was significantly upregulated at 7 dpi, whereas additional signatures related to antiviral sensing and immunity were significantly upregulated by 14 dpi (Figure 10B). In particular, upregulation of the antiviral signatures was statistically significant only at 14 dpi in the frontal cortex (Figure 10B). Additional pathways on 7 and 14 dpi in the brain of rhesus macaques revealed that at 7 dpi, several metabolic pathways and pathways of T lymphocyte activation and migration were activated (Supplemental Figure 3). In contrast, at 14 dpi, pathways related to leukocyte activity and pathways of antigen-presenting cells, T lymphocytes, neuroinflammation, and antiviral response and immunity were upregulated in the brain (Supplemental Figure 4).

### T lymphocyte dynamics in the brain with acute SIV infection.

**Figure 11:**
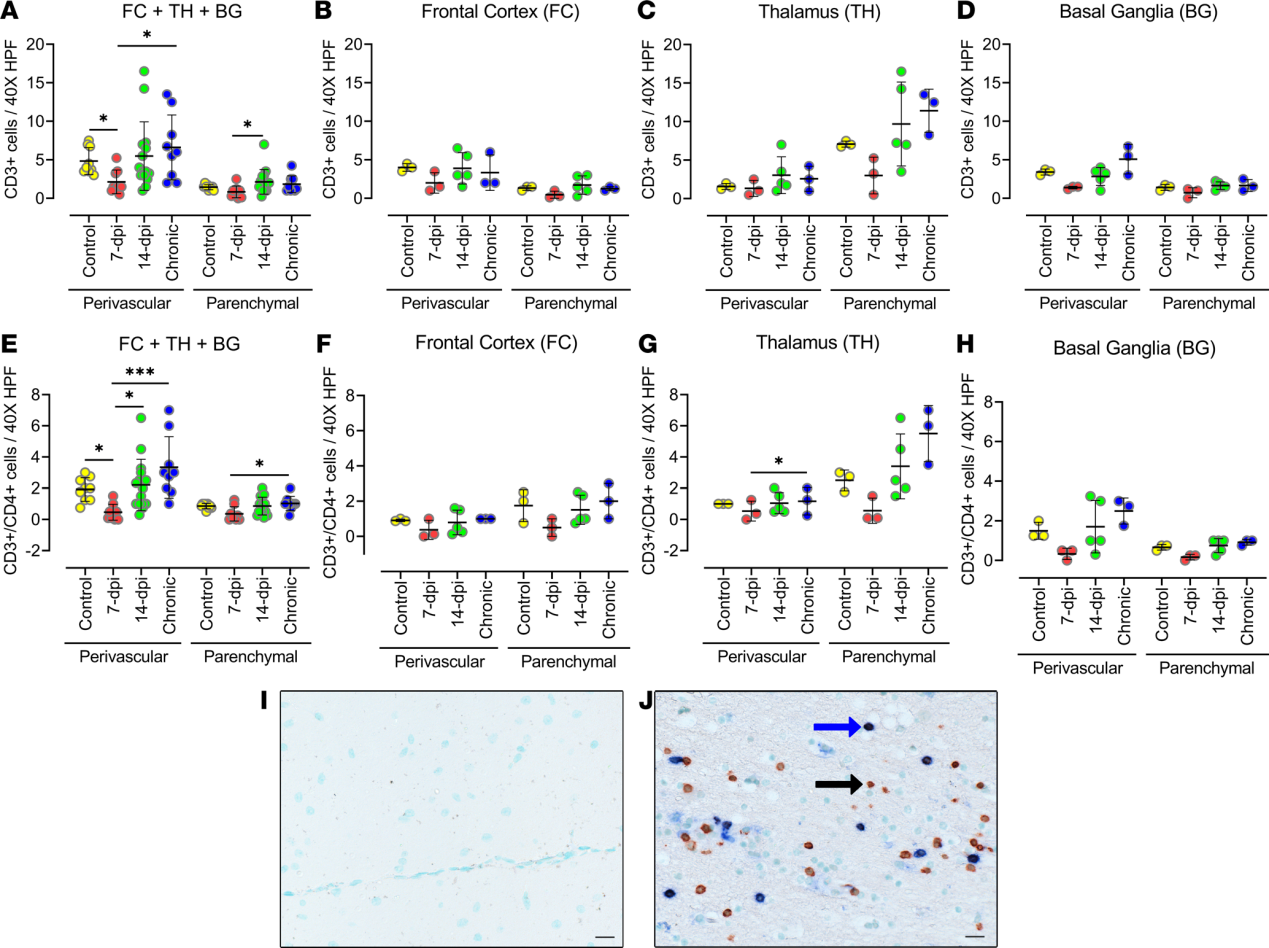
The number of CD3^+^ (CD4^−^) and CD3^+^CD4^+^ T lymphocytes decreases in SIV-infected macaque brains during the first week of infection. (**A**–**H**) The CD3^+^ (CD4^−^) (**A**–**D**) and CD3^+^CD4^+^ (**E**–**H**) T lymphocytes in frontal cortex, basal ganglia, and thalamus of SIV-infected macaque brain at 7 and 14 dpi. The sections were stained through double IHC staining using anti-CD3 and anti-CD4 antibodies. Analysis of double-positive cells in the 3 brain regions combined (**A** and **E**) and in individual brain regions: frontal cortex (**B** and** F**), thalamus (**C** and** G**), and basal ganglia (**D** and** H**). Each data point indicates 1 animal, and the associated numerical value is the average number of cells counted in 15 HPFs at 40× under light microscope. (**I** and **J**) IHC images of negative control (**I**) and positive staining (single stained [black arrow] for CD3 in brown; double stained [blue arrow] for CD3 and CD4 in brown and blue, respectively) (**J**). Data are presented as mean ± SD. Statistical significance was calculated using a nonparametric Kruskal-Wallis test and multiple comparisons were assessed using Dunn’s post hoc analysis. *P ≤ 0.05, ***P ≤ 0.001. *n* =3 (control, 7 dpi, chronic), *n* = 5 (14 dpi) (**A**–**H**). Scale bar: 20 μm (**I** and** J**).

To determine T lymphocyte infiltration into the brain during acute SIV infection, we performed IHC analysis (Figure 11). The IHC analysis of CD3^+^ T lymphocyte populations in all 3 brain regions combined showed a significant decrease in CD3^+^ T lymphocytes in the perivascular location at 7 dpi (*P* = 0.041) when compared with the control. This was followed by an increasing trend at 14 dpi and a significant increase in the chronically infected macaque (*P* = 0.0233) when compared with 7 dpi (Figure 11A). Likewise, the chronically infected animals had a similar number of CD3^+^ T cells at 14 dpi as the control animals but a significantly higher number than animals at 7 dpi. Interestingly, all 3 brain regions followed a similar T lymphocyte response pattern to infection despite showing a difference in quantities of SIV^+^ cells (Figure 11, B–D). Each of the 3 brain regions showed a trend of decreasing quantities of CD3^+^ cells at 7 dpi followed by an increase in those cells at 14 dpi, and CD3^+^ cell numbers at 14 dpi were not significantly different from the numbers present in the negative control group or the numbers in the chronic infection group. An exception was the parenchymal locations of the thalamus, where all groups, except the 7 dpi group, contained more CD3^+^ T cells (nearly double the control CD3^+^ T cell count) than the corresponding group at perivascular locations, although it did not statistically differ, most likely because of the small number of animals in the group.

To further define the CD3^+^ cells, we performed costaining to detect CD3^+^ and CD4^+^ T lymphocytes. The CD4^+^ T lymphocytes also followed the same pattern of immune response as CD3^+^ T lymphocytes (Figure 11, E–H). Although CD8^+^ T lymphocytes were not stained directly, the CD3^+^/CD4^−^ T lymphocytes similarly followed the same pattern of response to infection. The CD3^+^/CD4^+^ T lymphocytes comprised 20% to 40% of CD3^+^ T lymphocytes, implying that a large population of CD3^+^ T lymphocytes could be CD8^+^ T cells (CD3^+^CD4^−^ T lymphocytes) (Figure 11, I and J). Further supporting the significant role played by CD8^+^ cells in the brain, our RNA-Seq data also showed that signatures of CD8^+^ T lymphocytes were more upregulated than the signatures of the CD4^+^ T lymphocytes in the frontal cortex of the macaque brain at 14 dpi (Figure 7B).

## Discussion

The dynamic nature of CNS immune responses during hyper-acute SIV infection remains poorly understood. Here, we reported on an examination of the early immune activation and associated sequelae of events in the CNS after acute SIV_mac239_ infection in rhesus macaques. Coupling histological analysis with RNA-Seq data, we demonstrated immune and inflammatory responses that may have created an early environment favorable for virus survival in the brain.

An active proinflammatory response in the brain, evidenced by detection of IFN-α and IL-6 signaling markers, was detected at 7 dpi, but the virus was not yet reliably detected in the tissue until 14 dpi. We hypothesize that at the onset of viral infection in the periphery, the initial upregulation of proinflammatory factors (IL-6, type 1 and 2 IFNs) may have altered the endothelial cells in the blood brain barrier, favoring the entry of proinflammatory cytokines and/or lymphocytes, leading to microglia and neuronal activation, even prior to virus entry (22–24). Such a proinflammatory environment is very likely an early mechanism to prevent virus survival in the brain.

The increased proinflammatory immune milieu in the brain was followed by antiinflammatory responses. The increased TGF-β expression at 14 dpi was coupled with the rise of the number of macrophages, especially in the perivascular location, indicating a potential association of virus-induced antiinflammatory responses in these cells, corroborating a previous ex vivo study demonstrating that HIV virions produced in macrophages, and not those derived from T cells, express the CD36 receptor, which induces TGF-β production by the thrombospondin 1 (TSP-1) ligand (25). Thus, the delayed TGF-β expression in macrophages until 14 dpi could be due to the delay of viral entry, given that we colocalized the virus to macrophages only in this cohort. By carrying the virus and expression of TGF-β at the same time, macrophages inadvertently ensured viral survival in the proinflammatory environment in this early acute infection model. Additionally, the upregulation of IL-10, STAT3, and SOCS3 gene signatures in the brain also suggest the existence of a negative feedback loop mechanism based on the IL-10/STAT3/SOCS3 axis to counter the intrathecal inflammation (26). Although the antiinflammatory response early in the brain may be protecting tissue from inflammation, it was not sustained in chronically infected animals, and thus unable to counter the long-term consequences of inflammation.

During acute infection, depletion of CD4^+^ T cells occurs in the periphery and tissues, and CD8^+^ T cells accumulate in the CNS during chronic infection (27–29). To our knowledge, CD3^+^ T cell dynamics in acute infection have not been previously reported. The current study, based on IHC analysis, revealed that CD3^+^ T cells (CD4^+^ and CD8^+^ T cells) were depleted in SIV-infected macaque brains in the first week of infection, but were replaced by the second week after infection. This homing of CD3^+^ T cells to the brain during the second week may occur at the CXCR3-CXCL10/CXCL11 axis, as evident from the several-fold upregulation of their corresponding gene signatures only during the second week (30, 31). Again, to our surprise, the aforementioned T cell dynamics were uniform among all 3 brain regions, supporting the hypothesis that this response could have been induced by peripheral factors. Furthermore, the infiltration had only replaced the initial loss of cells to the level of the uninfected control. But a higher number of CD8^+^ T cells among the total number of infiltrated T cells during the second week after infection in conjunction with upregulation of granzyme A and B transcripts — potentially also reflecting NK cell activation — suggest the cytotoxic nature of this response (31). These hypotheses require further validation.

Notwithstanding the above findings, we were limited by resources for carrying out RNA-Seq studies using the basal ganglia and thalamus, which could have added more information on the comparative tissue expression profile at the tested time points. In addition to macrophages, the upregulation of NK cells, DC signatures, and STAT3 signaling (key regulator of DC function) hints at their possible influence on immune response in the macaque brain during acute SIV infection (32). To our knowledge, there is a paucity of literature on their role in acute SIV infection. Further coordinated studies on NK cells and DCs along with other innate and adaptive immune cells could shed more light on the events that occur during early acute SIV infection in the brain.

In summary, to our knowledge, this is the first study on coordinated assessment of brain and peripheral immune activation coupled with the resident and infiltrating immune cells in the rhesus macaque brain during hyper-acute SIV infection. The detailed analysis revealed that although immune activation in the brain occurred before detectable viral load, a more precise antiviral response required detectable viral presence. Although the proinflammatory (IL-6) response initiated during acute infection persists over time, the antiinflammatory (TGF-β) response was short lived. By providing a clearer picture of acute events, our data have important implications in furthering the understanding of early immune response in the brain.

## Methods

### Animals and viral infection.

A cohort of 11 Indian-origin adult male rhesus macaques (*Macaca mulatta*) were used in the present study. Animals were housed and cared for at Biomere under the rules and regulations of the Committee on the Care and Use of Laboratory Animal Resources and in agreement with the American Association for Accreditation of Laboratory Animal Care standards and *Guide for the Care and Use of Laboratory Animals* (National Academies Press, 2011). Animals were fed a standard monkey chow diet supplemented daily with fruit and vegetables and water ad libitum. Social enrichment was delivered and overseen by veterinary staff and overall animal health was monitored daily. Animals were humanely euthanized using an overdose of barbiturates according to the guidelines of the American Veterinary Medical Association. When necessary, macaques were immobilized with ketamine HCl (Parke-Davis) at approximately 10 mg/kg and i.m. injected. Blood samples were collected using venipuncture. SIV_mac239X_ stock was received as a gift from Brandon Keele (AIDS and Cancer Virus Program, Frederick National Laboratory). Macaques were infected intrarectally with a single high-dose challenge of SIV_mac239X_ as described previously (16), and then 3 macaques were euthanized at 7 dpi, and 5 macaques were euthanized at 14 dpi. Chronically infected macaques were euthanized at more than 180 dpi. An additional 3 macaques served as uninfected controls for the study.

### Tissue collection and processing.

On the day of euthanization, blood was collected from each animal. Blood was processed on the same day for PBMC isolation, and the plasma was cryopreserved for viral load quantification at a later time. Brain was harvested and a tissue sample (roughly 1 cm × 1 cm × 1 cm) was collected from the frontal cortex from one of the hemispheres and cryopreserved in RNAlater for assessing viral load and for RNA-Seq analysis. For macaque Rh11, tissue samples from the basal ganglia and thalamus were also cryopreserved in RNAlater. The other hemisphere was stored in 10% neutral buffered formalin for 14 days, after which the hemisphere was sliced and tissue samples were collected from different brain regions, embedded in paraffin, and sectioned at 10 μm thickness for ISH and IHC studies. A tissue section from each brain region was stained with H&E for routine histological analysis.

### Peripheral viral load quantification.

Quantified RNA was used for RT-PCR against a conserved region of *gag* using gene-specific primers (33). In the first step, RNA was reverse-transcribed followed by treatment with RNase for 20 minutes at 37°C. Next, cDNA was amplified using 7300 ABI Real-Time PCR system (Applied Biosystems) according to the manufacturer’s protocol (TaqMan Fast Advanced Master Mix User Guide; publication 4444605).

### Brain viral load quantification — DNA + RNA.

Viral loads were quantified from frozen brain tissue as previously described (34). Briefly, genomic DNA was extracted using a QIAamp 96 QIAcube HT kit and total RNA was extracted using an RNeasy 96 QIAcube HT kit according to the manufacturer’s specifications (Qiagen). Quantitative PCR was used to measure tissue-associated SIV DNA and RNA levels. After sample processing, total RNA was reverse-transcribed using Superscript III VILO (Invitrogen) according to the manufacturer’s specifications. Standards were used to calculate SIV Gag DNA and RNA copies. In order to generate RNA standards, the AmpliCapMax T7 High Yield Message Maker kit (Cell Script) followed by downstream RNA purification using an RNA clean and concentrator kit (Zymo Research) were used. Log dilutions of the standards were included with each RT-PCR assay and an RPP30 control was included in the SIV DNA PCR assays. RT-PCR assays were run in duplicate and viral DNA assays were run in triplicate on the Quantstudio 6 Flex system (Applied Biosystems) using the following thermocycler settings: 95°C for 20 seconds for initial denaturation and 95°C for 1 second followed by 60°C for 20 seconds repeated for 45 cycles. The following SIV Gag-targeting primers and probes were used for PCR assays: sGag21 (forward) GTCTGCGTCATCTGGTGCATTC, sGag22 (reverse) CACTAGGTGTCTCTGCACTATCTGTTTTG, and sGag23 (probe) 5′FAM-CTTCCTCAGTGTGTTTCACTTTCTCTTCTGCG-BHQ-3′. Additionally, a commercially available RPP30 gene-specific TaqMan assay was used (Thermo Fisher Scientific, Hs00922551_cn). Tissue viral loads in SIV Gag DNA copies per million cells were calculated using the RPP30 gene to normalize for cell input. The tissue SIV DNA assay limit of detection (LOD) was 8 copies per million cells. Tissue SIV RNA viral loads were calculated as RNA copies per μg of total RNA input. Tissue SIV RNA assay LOD was 1 copy per μg of total RNA input.

### In situ hybridization for SIV RNA.

SIV RNA detection in macaque brain tissue samples (frontal cortex, basal ganglia, and thalamus) was performed using an RNAscope assay kit from Advanced Cell Diagnostics. Custom probes for SIV_mac239_ were used in conjunction with RNAscope 2.5 HD Detection kit (Brown) according to the manufacturer’s instructions. To detect/quantify the number of SIV^+^ cells, the entire section was scanned frame by frame for the presence of probe-positive cells.

### IHC.

IHC detection of immune cell lineage markers (CD3, CD163/CD68) and glial cell markers (GFAP, Iba-1) was performed by indirect IHC. Double or single IHC staining of immune cells was carried out as follows. Briefly, FFPE sections were deparaffinized and then rehydrated through xylene and graded alcohol series. To quench endogenous peroxidase activity, slides were then incubated with 3% H_2_O_2_ for 5 minutes and antigen retrieval was performed using AUS citrate buffer (Vector Laboratories) in a steam chamber for 60 minutes. Sections were then blocked with 2.5% normal horse serum (Vector Laboratories) for 20 minutes followed by incubation with appropriate primary antibody overnight at 4°C. DAKO protein block (Agilent) was used to dilute primary antibodies: CD3, 1:400 (clone UCHT1, R&D Systems); CD68, 1:200 (clone 298807, R&D Systems); GFAP, 1:500 (polyclonal rabbit, Dako, Z0334); Iba-1, 1:1000 (polyclonal rabbit, Wako, 019-19741); CD163, 1:400 (clone EDHu1, Bio-Rad). Appropriate IgG controls were run during each assay. Secondary antibody conjugated ImmPRESS peroxidase (HRP) (Vector Laboratories) polymer reagent was added, and the sections were incubated for 30 minutes. Sections were then exposed to ImmPACT peroxidase substrate until optimal color intensity was achieved. For experiments involving double labeling, sections were again blocked with 2.5% normal horse serum and the process was repeated with appropriate primary antibody incubation; otherwise, the sections were counterstained with Mayer’s hematoxylin (Sigma-Aldrich) and mounted using VectaMount mounting medium. The double staining was carried out using secondary antibody conjugated to alkaline phosphatase (AP) coupled with respective AP detection substrate (Vector Laboratories). The double-stained sections were counterstained with methyl green (Vector Laboratories, H-3402) and mounted with VectaMount mounting medium.

Proinflammatory IL-6 (1:500, polyclonal rabbit, LSBio, LS-A9692) and antiinflammatory TGF-β (1:1000, clone TB21, LSBio) were colocalized with cell identification markers (CD3, CD163/CD68, Iba-1, GFAP) on FFPE brain tissue sections using the double immunostaining method above to characterize cells expressing respective inflammatory markers. The double-positive cells were counted as described below using a bright-field microscope. Additionally, the CD3^+^ T cells were classified as CD3^+^/CD4^+^ or CD3^+^/CD4^–^ using the same anti-CD3 antibody as above and anti-CD4 antibody at 1:200 (clone EPR6855; Abcam) through double staining as described earlier.

Bright-field microscopy was performed using Zeiss Axio Imager M1 microscope and 10 to 20 high-powered fields (HPFs) were captured at either 20× or 40× and cells were counted. For CD3^+^, CD4^+^, and CD163^+^/CD68^+^ cells and for double staining, the number of single- or double-positive cells was also quantified separately based on their location; whether lining the blood vessels, perivascular (V), or in the brain parenchyma (P). Cell counts were then averaged across the number of images captured (15 frames/section).

### Dual labeling — IHC and ISH.

For experiments involving immunolabeling for either CD3 or CD163 and ISH for SIV, IHC was first performed. Sections were then incubated with Protease Plus (provided in RNAscope kit) for 30 minutes and blocked with 10% normal horse serum for 30 minutes. This was followed by primary and secondary antibody incubation and development of antigen signal using ImmPACT peroxidase substrate kit. Following this, ISH was started with 2-hour incubation with probes for SIV and proceeded as per the manufacturer’s instructions for RNAscope Multiplex Fluorescent Reagent kit v2 (ACD) coupled with PerkinElmer TSA Plus Fluorescein system. The sections were incubated with DAPI (ACD) before mounting with VectaMount mounting medium.

Confocal microscopy was performed using Zeiss LSM 880 laser scanning confocal microscope, and 20 HPFs were captured at either 40× or 80× and cell analysis was performed.

### Quantitative image analysis.

Quantitative image analysis was performed to quantify IHC expression of GFAP and Iba-1 using HALO software (v2.3.2089.27 or v3.0.311.405; Indica Labs) on representative 50 mm^2^ areas in histological sections in the 3 brain regions (frontal cortex, thalamus, and basal ganglia) using the Area Quantification v2.1.7 module, tuning the algorithm to determine the percentage and area of tissue with positive IHC expression of GFAP and Iba-1. After the quantitative image analysis was performed, manual inspection was performed in each sample to ensure that the positively and negatively annotated areas were accurate.

### Library preparation and Bulk RNA-Seq.

RNA was isolated from the frontal cortex tissue preserved in RNAlater using the RNeasy Mini kit (Qiagen) with on-column DNase digestion. RNA quality was assessed using an Agilent Bioanalyzer, and 10 ng of total RNA was used as input for cDNA synthesis using the Clontech SMART-Seq v4 Ultra Low Input RNA kit (Takara Bio) according to the manufacturer’s instructions. Amplified cDNA was fragmented and appended with dual-indexed barcodes using the NexteraXT DNA Library Preparation kit (Illumina). Libraries were validated by capillary electrophoresis on an Agilent 4200 TapeStation, pooled at equimolar concentrations, and sequenced on an Illumina HiSeq3000 at 100SR, yielding 20–25 million reads per sample. Alignment was performed using STAR version 2.5.2b (35) and transcripts were annotated using the MacaM genome build. Transcript abundance estimates were calculated internal to the STAR aligner using the algorithm of HTSeq-count (36). DESeq2 was used for normalization (37), producing a normalized read count table and a regularized log expression table. DESeq2 was also used to examine per-gene differential expression between groups using its standard methods for the Wald test. Genes that were significantly differentially expressed by a Benjamini-Hochberg adjusted *P* value less than 0.05 were examined for significant overlap with gene sets from the “chemical and genetic perturbations,” “canonical pathways,” and “GO biological process” collections of the Broad Institute Molecular Signatures Database using Fisher’s exact test. Functional analysis of statistically significant gene and protein changes was performed using Ingenuity Pathways Analysis (IPA; Qiagen). For all GSEA, a right-tailed Fisher’s exact test was used to calculate *P* values associated with each biological function and canonical pathway. The calculated *z* score signifies whether gene or protein expression changes for known targets of each regulator are consistent with what is expected from the literature (*z* > 2, regulator predicted to be activated, *z* < –2, regulator predicted to inhibited). Sample-level enrichment score (SLEA) represents the mean expression of all significant genes within each pathway for each individual animal. An SLEA *z* score greater than 0 corresponds to a pathway for which member genes are upregulated, and an SLEA *z* score inferior to 0 corresponds to a pathway with genes downregulated in that sample. Columns correspond to individual animals, and rows correspond to individual pathways scaled across all animals using the *z* score R function.

### Statistics.

Statistical analyses were performed using GraphPad Prism 9.0, unless otherwise mentioned. The data were analyzed with a nonparametric Kruskal-Wallis test and multiple comparisons were assessed using Dunn’s post hoc analysis. Unless otherwise stated, data are presented as mean ± SD. *P* values (multiplicity-adjusted *P* values for multiple comparison) less than 0.05 were considered significant.

### Study approval.

All procedures in the animal study were approved by the Biomere IACUC under protocols 17–13.

## Author contributions

CST, RKR, and MA designed this study. RMG, MA, NBM, CD, SM, EG, VV, RJ, SEB, CPM, and AJM performed the experiments. RMG, MA, CD, and SM carried out the data analysis. RMG, SM, RKR, and CST wrote the manuscript, and all others edited the manuscript.

## Supplementary Material

Supplemental data

## Figures and Tables

**Table 1:**
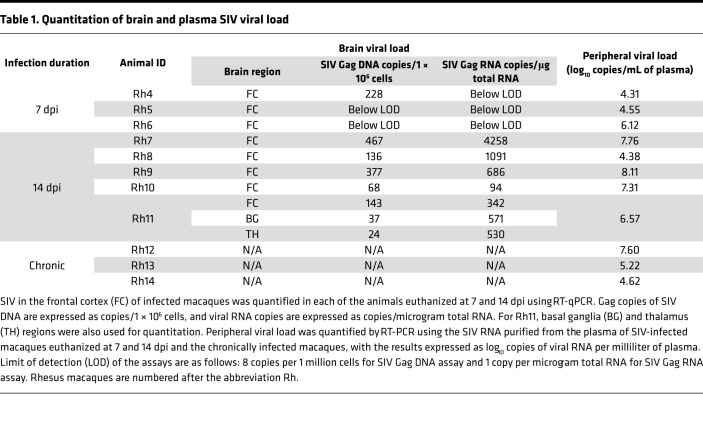
Quantitation of brain and plasma SIV viral load

## Version 1 10/22/2021

Electronic publication
