# Supplementary material for: Increased IL-6 expression precedes reliable viral detection in the rhesus macaque brain during acute SIV infection
Source: JCI Insight. 2021 Oct 22;6(20):e152013. doi: 10.1172/jci.insight.152013 (PMC8564899; doi:10.1172/jci.insight.152013)
Supplement: Supplemental data [file jciinsight-6-152013-s241.pdf]

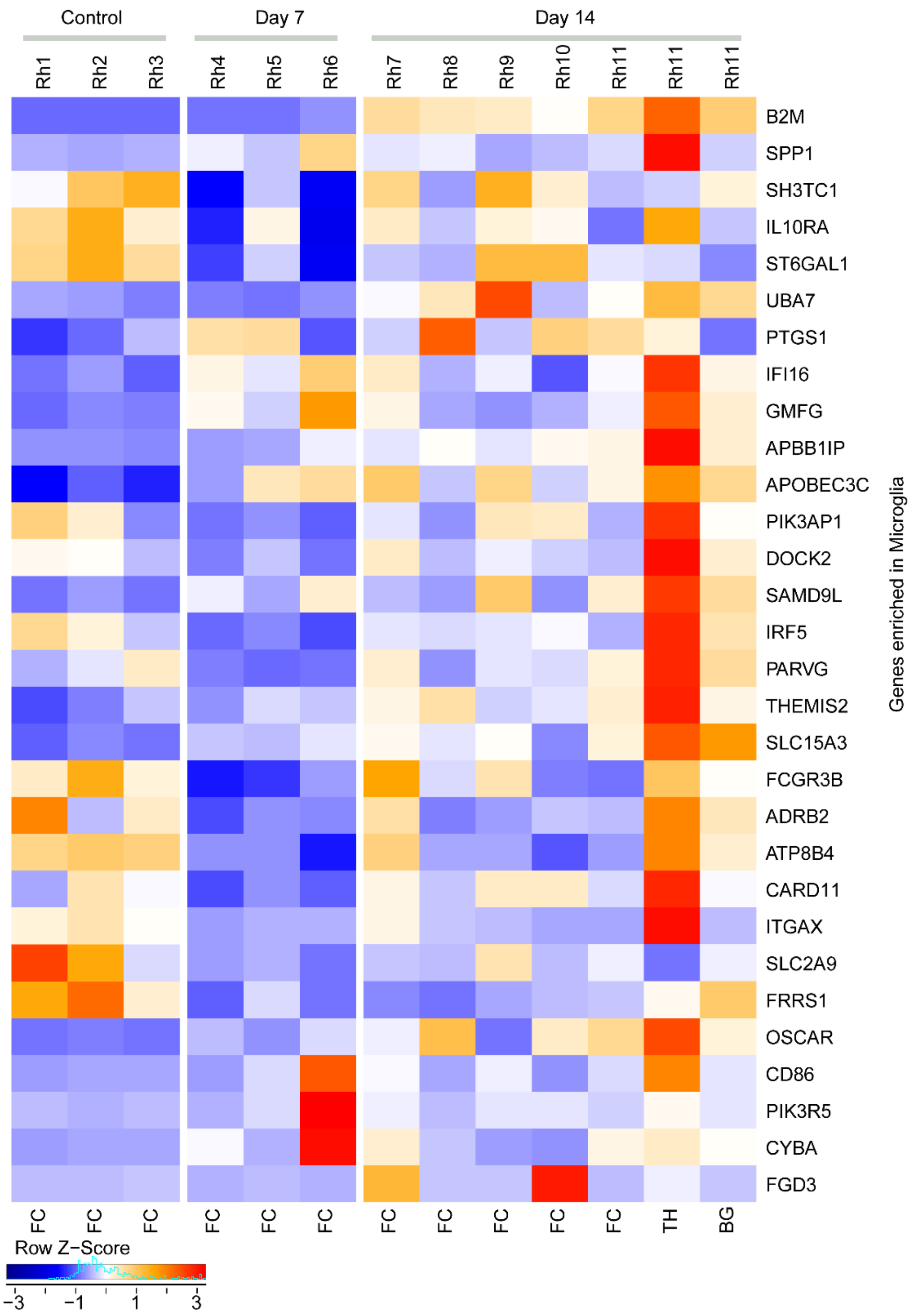

Supplemental Figure 1. Enrichment of microglia gene signatures in SIV infected macaque brain. Expression of a microglia gene signatures in the frontal cortex (FC) of SIV infected macaque at 7 and 14 dpi compared to uninfected control animals. For Rh11, basal ganglia (BG) and thalamus (TH) regions were also used. Only genes that were up or down regulated after correcting for multiple testing (Adjusted BH *P* value < 0.05) were shown on the heatmap. Each row represents a gene normalized mRNA expression across all animals. Each column represents an animal belonging to control group (first bloc) and at day 7 (second bloc) and day 14 (third bloc) following SIV infection. Changes in the Row *Z*-score are represented as unique color from lowest (blue) to highest (red) color gradient. *n*=3 (control, 7 dpi), *n*= 5 (14 dpi).

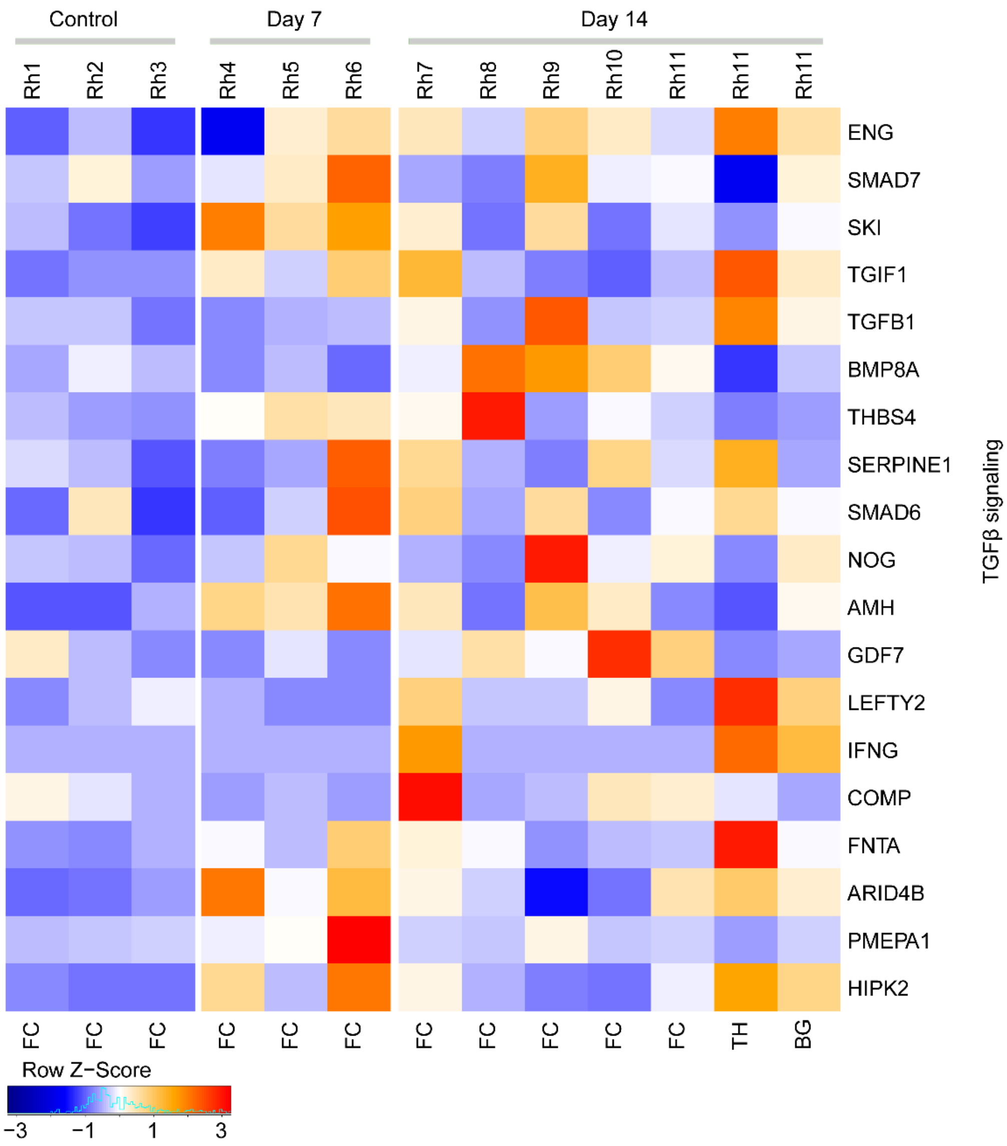

Supplemental Figure 2. Enrichment of TGF-β gene signatures in SIV infected macaque brain. Expression of a TGF-β gene signatures in the frontal cortex (FC) of SIV infected monkeys at 7 and 14 dpi compared to uninfected control animals. For Rh11, basal ganglia (BG) and thalamus (TH) regions were also used. Only genes that were up or down regulated after correcting for multiple testing (Adjusted BH *P* value < 0.05) were shown on the heatmap. Each row represents a gene normalized mRNA expression across all animals. Each column represents an animal belonging to control group (first bloc) and at day 7 (second bloc) and day 14 (third bloc) following SIV infection. Changes in the Row *Z*-score are represented as unique color from lowest (blue) to highest (red) color gradient. *n*=3 (control, 7 dpi), *n*= 5 (14 dpi).

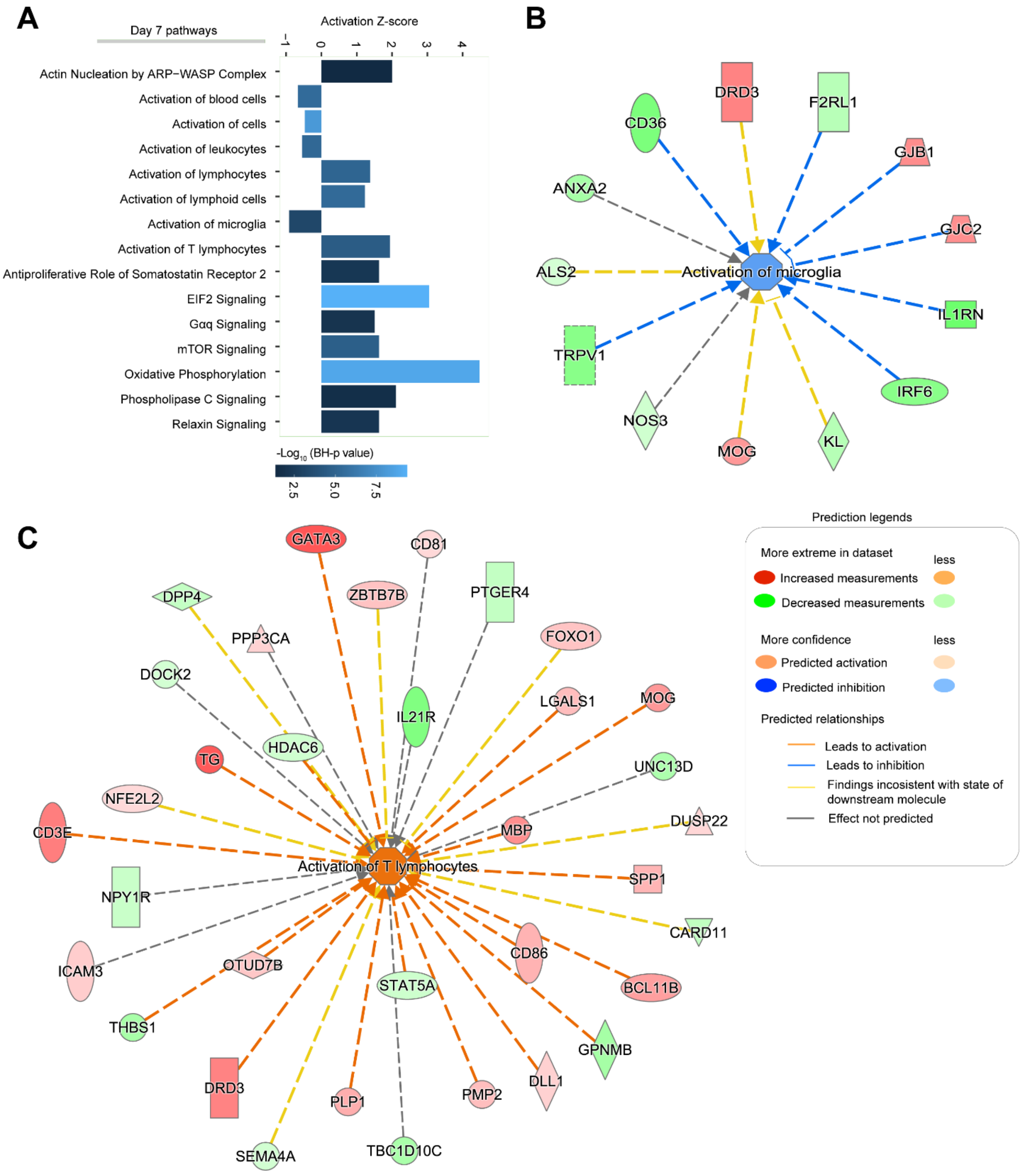

Supplemental Figure 3. Additional pathways of activation and metabolism at day 7 post-infection in SIV infected macaque brain. (**A**–**C**) Additional pathways of metabolism, mTOR signaling and other signaling pathways in the frontal cortex of SIV infected macaque at 7 dpi (**A**). Network map of microglial activation (**B**) and T-lymphocytes activation (**C**) with corresponding gene signatures that were increased (in red) or decreased (in green). Barplots color gradient represents the –$\log_{10}$ (*P* values) of each pathway as reported by ingenuity pathway analysis (IPA). Barplots represent the enrichment score (*Z*-score) of each pathway (**A**). Networks show genes from selected pathways (**B** and **C**).

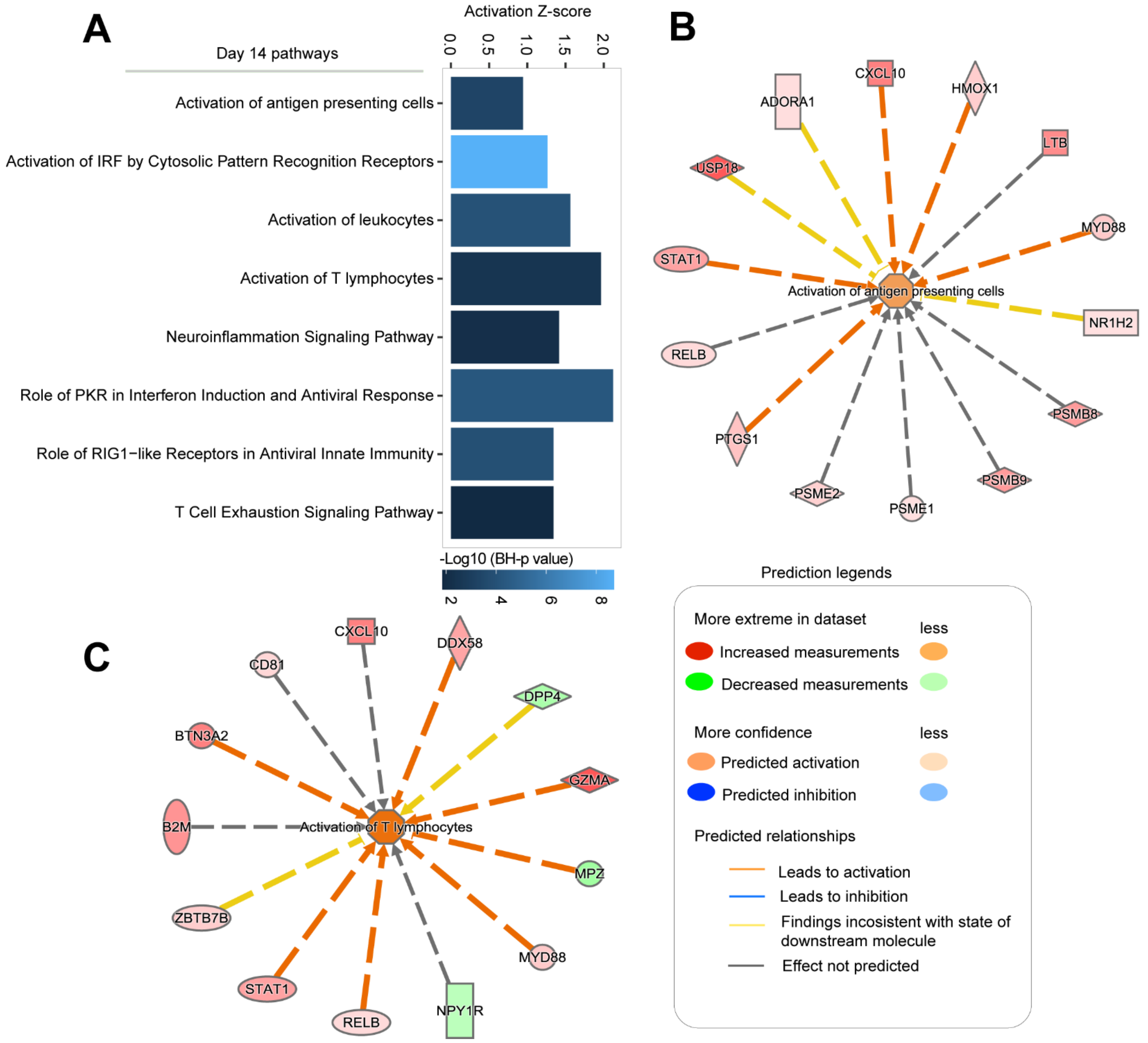

Supplemental Figure 4. Additional pathways of activation and metabolism at day 14 post-infection in SIV infected macaque brain. (**A**–**C**) Additional pathways of metabolism and other signaling pathways in the frontal cortex of SIV infected macaque at 14 dpi (**A**). Network map of antigen presenting cells activation (**B**) and T-lymphocytes activation (**C**) with corresponding gene signatures that were increased (in red) or decreased (in green). Barplots color gradient represents the $-\log_{10}$ (*P* values) of each pathway as reported by ingenuity pathway analysis (IPA). Barplots represent the enrichment score (*Z*-score) of each pathway (**A**). Networks show genes from selected pathways (**B** and **C**).
